# Preclinical evaluation of topically-administered PEGylated Fab’ lung toxicity

**DOI:** 10.1016/j.ijpx.2019.100019

**Published:** 2019-06-21

**Authors:** Danielle Freches, Natacha Rocks, Harshad P. Patil, Fabienne Perin, Jacques Van Snick, Rita Vanbever, Didier Cataldo

**Affiliations:** a Advanced Drug Delivery & Biomaterials, Louvain Drug Research Institute, Université catholique de Louvain (UCLouvain), Brussels, Belgium; b Laboratory of Tumor and Development Biology, GIGA-Cancer and GIGA-I^3^, GIGA-Research, University of Liege, Liege, Belgium; c Ludwig Cancer Research Ltd, Brussels Branch, Avenue Hippocrate 74, UCLouvain, 7459, B-1200 Brussels, Belgium; d Department of Respiratory Diseases, University of Liege and CHU Liege, Liege, Belgium

**Keywords:** Pulmonary drug delivery, Monoclonal antibodies, PEGylation, Lung toxicity, Alveolar macrophages

## Abstract

PEGylation is a promising approach to increase the residence time of antibody fragments in the lungs and sustain their therapeutic effects. However, concerns arise as to the potential pulmonary toxicity of antibody fragments conjugated to high molecular weight (HMW) polyethylene glycol (PEG), notably after repeated administrations, and the possibility of PEG accumulation in the lungs. The purpose of this proof-of-concept study is to give insights about the safety of lung administration of a Fab’ anti-IL17A antibody fragment conjugated to two-armed 40 kDa PEG (PEG40). The presence of the PEG40 moiety inside alveolar macrophages remained stable for at least 24 h after intratracheal administration of PEG40-Fab’ to mice. PEG40 was then progressively cleared from alveolar macrophages. Incubation of PEG40 alone with macrophages *in vitro* did not significantly harm macrophages and did not affect phagocytosis or the production of inflammatory markers. After acute or chronic administration of PEG40-Fab’ to mice, no signs of significant pulmonary toxicity or inflammatory cell accumulation were observed. A vacuolization of alveolar macrophages not associated with any inflammation was noticed when PEG40, PEG40-Fab’, or unPEGylated Fab’ were administered. To conclude this preliminary proof of concept study, acute or repeated pulmonary administrations of PEGylated Fab’ appear safe in rodents.

## Graphical abstract

**Figure:**
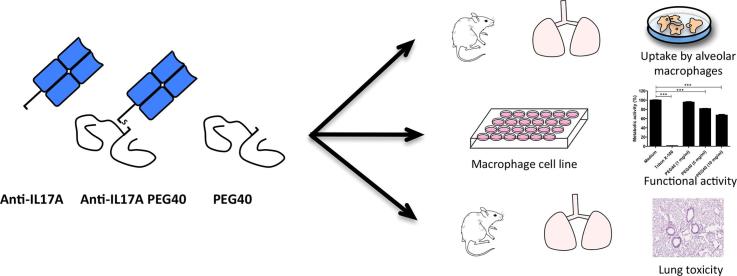

## 1 Introduction

Polyethylene glycol molecules (PEG) are synthetic, highly water soluble, inert polymers that can be produced in a wide range of molecular weights (MW). PEGs are largely used by the pharmaceutical industry with the purpose to improve serum half-life of molecules, increase their stability, and in some cases, decrease protein immunogenicity. Twelve PEGylated biopharmaceuticals have been marketed and many more are in preclinical or clinical development (Turecek et al., 2016).

Pre-approval toxicological studies of marketed products indicate that PEGs exhibit a low toxicity profile. Reported toxic effects of such pharmaceutical compositions are thought to be caused by the active part of the drug molecule rather than by the PEG moiety (Ivens et al., 2015, Jevsevar et al., 2010, Kang et al., 2009, Webster et al., 2007). The only consequence attributed to the sole PEG administration reported to occur with approximately half of the approved PEGylated drugs is a macrophage vacuolization found in a variety of tissues (e.g. spleen, lymph nodes, choroid plexus, thymus, lungs) and apparently not linked to macrophage dysfunction (Ivens et al., 2015).

The pulmonary route is an attractive and alternative way of administration for drugs and biotherapeutics to target respiratory diseases with the advantages of high drug concentrations being available locally in lung parenchyma, and low side effects since the systemic dose is maintained very low. In this context, PEGylation represents a promising approach to sustain the presence of biopharmaceuticals in the lungs and to enhance their overall therapeutic efficiency (Guichard et al., 2017a). Recently, using preclinical animal models, others and our groups have reported the feasibility and the efficacy of the pulmonary administration of PEGylated compounds, such as Fab’ (fragment antigen-binding) and biopharmaceutical and chemotherapeutic agents (Cantin et al., 2002, Freches et al., 2017, Koussoroplis et al., 2013, Koussoroplis et al., 2014, Luo et al., 2016, Mcleod et al., 2015). However, the safety of administering PEG or PEGylated compounds directly to the lungs by inhalation and the potential impact on the pulmonary tissue has not been studied in an extensive manner yet. While low molecular weight (LMW) PEGs (<10 kDa) are considered safe and are commonly used as excipients in nasal and inhaled formulations, the use of larger PEGs (as up 40 kDa for biopharmaceuticals) have raised safety concerns about their potential pulmonary toxicity. Indeed, PEG could hypothetically induce a pulmonary inflammation on long-term use while the potential retention of the PEG inside alveolar macrophages could be associated to adverse effects on cell functions. Noteworthy, the accumulation of PEGs in macrophages could be an important issue as the administration of PEGylated antibodies in a chronic manner is expected.

In the present study, the residence time of high molecular weight (HMW) PEG40 in alveolar macrophages was analyzed *in vivo* and their effects on macrophage functions were evaluated *in vitro*. In addition, the pulmonary toxicity of a monoPEGylated Fab’ anti-IL17A (PEG40-Fab’ anti-IL17A or PEG40-Fab’) administered by the intratracheal route was assessed in acute and chronic studies in mice.

## 2 Material and methods

### 2.1 Reagents

Two-armed 40 kDa PEG maleimide was purchased from NOF Corporation (Japan). For *in vivo* and *in vitro* experiments on PEG alone, the maleimide function was neutralized by a thiol conjugation. For that, PEG40-maleimide was treated with a large excess (50-fold molar excess) of β-mercaptoethylamine (Sigma Aldrich) in 0.1 M sodium phosphate buffer, pH6.2, overnight at room temperature under agitation. The solution was then dialyzed 3 times in phosphate buffered saline (PBS) (Lonza) to remove the excess of β-mercaptoethylamine.

The murine Fab’ anti-IL17A containing a single free cysteine at the hinge region to react selectively with one molecule of PEG by thiol PEGylation was provided by UCB Pharma (United Kingdom). The Fab’ was conjugated to one molecule of a two-armed 40 kDa PEG (abbreviated as PEG40-Fab’ anti-IL17A or PEG40 Fab’) by thiol-directed PEGylation, as previously reported (Freches et al., 2017). The attachment of one PEG chain per Fab’ fragment was confirmed by molecular weight analysis using sodium dodecyl sulfate polyacrylamide gel electrophoresis and matrix-assisted laser desorption/ionization time-of-flight mass spectrometry (Freches et al., 2017). PEG40, Fab’ and PEG40-Fab’ preparations were tested for LPS contamination using the Endpoint Chromogenic LAL assay (Lonza) according to manufacturer’s protocol.

### 2.2 Cell culture

J774A.1 cells were chosen as a model macrophage-like cell line due to their constant and rapid growth rate, and suitability as comparator cell line for *in vivo* alveolar macrophage responses observed in a murine BALB/c model (Forbes et al., 2014). Cells were cultured in Dulbecco’s Modified Eagle Medium (DMEM; Gibco) supplemented with 10% fetal bovine serum (v/v) (FBS; Gibco) and antibiotics (100 µg/ml streptomycin and 100 units/ml penicillin) in a humidified atmosphere of 5% CO_2_ at 37 °C. Cells were subcultured when they reached 70–80% confluence. J774A.1 cells were exposed to 1, 5 or 10 mg/ml of PEG40.

### 2.3 In vitro cytotoxicity and cell viability

J774A.1 cells were cultured at a density of 2,000 cells/well in a 96-well plate overnight. Cells were then exposed to different concentrations of PEG40 (1 mg/ml, 5 mg/ml, 10 mg/ml). After 3 days of culture, plasma membrane integrity was evaluated by quantifying the release of lactate dehydrogenase (LDH) in the culture supernatant and treated cells were further used for the 3-(4,5-dimethythiazol-2-yl)-2,5-diphenyl tetrazolium bromide (MTT) assay to measure cell metabolic activities.

LDH activity was assessed using the Pierce LDH assay kit (Pierce). Results were expressed as the percentage of activity detected in the medium of each samples over the activity in the cells from the positive Triton X-100 group. Cell metabolism and cell viability were assessed by the MTT test (Sigma Aldrich). For this, PEG- or control-treated cells were washed with culture medium and incubated with 0.5 mg/ml MTT for 3 h to form insoluble blue formazan crystals. Dye intensity is proportional to the number of cells that are alive and that are not impaired regarding their metabolic activities. The blue crystals were solubilised with dimethyl sulfoxide (DMSO) and the optical density (OD) was measured at 560 nm. The metabolic activity was expressed as a percentage of the mean value recorded for untreated cells. In all assays, untreated cells and cells treated with Triton X-100 were used as negative and positive controls, respectively.

### 2.4 In vitro phagocytosis assay

J774A.1 cells were seeded at a density of 30,000 cells/well in a 48-well plate and cultured overnight. Cells were then exposed to different concentrations of PEG40 (1 mg/ml, 5 mg/ml, 10 mg/ml) diluted in complete culture medium. After 3 days, cell culture medium was removed and replenished with 500 μl of fresh cell culture medium containing 1 μm polystyrene beads (latex beads, carboxylate modified polystyrene fluorescent red, Sigma Aldrich) at a concentration of 2 × 10^8^ beads/ml and incubated for 4 h. Cells were then washed three times with 1 ml of PBS and lysed in 0.1% triton X-100 lysis buffer. The fluorescence of the cell lysates was measured with an excitation wavelength at 575 nm and emission at 610 nm. Data are presented as a percentage of the phagocytic activity of untreated J774A.1 cells.

### 2.5 In vitro macrophage response to LPS challenge

J774A.1 cells were seeded at a density of 30,000 cells/ well in a 48-well plate, cultured overnight and exposed during 3 days to different concentrations of PEG40 (1 mg/ml, 5 mg/ml, 10 mg/ml) diluted in complete medium. The medium was then removed and replaced with fresh medium ± LPS from *Escherichia coli* (1 µg/ml, Sigma Aldrich). Following an overnight incubation, supernatants were collected and nitric oxide production was determined in the form of total nitrites using the Griess reaction (Griess reaction kit, Biotium). IL-6 production was determined by a custom-made ELISA (Vink et al., 1990) and TNF-α was measured by using Mouse TNF alpha ELISA Ready-SET-Go kit (Affymetrix, eBioscience).

### 2.6 In vivo measurement of alveolar macrophages internalization and clearance of antibodies and PEG40

Experimental protocols were approved by the Institutional Animal Care and Use Committee of the Université catholique de Louvain (Permit number: 2012/UCL/MD/006) and by the animal ethical committee of the University of Liege (under the reference #1001). Specific-pathogen-free female NMRI mice (8 to 10-week old; Janvier Labs, France) were used. The study was performed under anaesthesia (intraperitoneal injection of ketamine/xylazine (90/10 mg/kg) and all efforts were made to minimize animal suffering. One hundred µg PEG40-Fab’ or Fab’ were administered by the intratracheal route to the mice (100 µg/25 µl PBS) (Freches et al., 2017). A laryngoscope (Penn-Century Inc., Philadelphia, USA) was used to correctly place the bended and blunt needle of a 100 µl precision micro-syringe (Hamilton, Bonaduz, Switzerland) into the trachea. Mice were placed on their back with a tilt angle of 45˚. Twenty-five µl of solution containing 100 µg of PEG40-Fab’ or Fab’ were delivered followed by a 200 µl air bolus using a 1 ml syringe having an angled 18 gauge needle with a blunt tip in order to push the solution to the distal tract. PEG was not counted in the mass delivered and the two mice groups therefore received 2.1 nmol of Fab’ per mouse.

Mice were sacrificed by cervical dislocation at 4 h, 24 h, 7 days and 14 days post-administration. Bronchoalveolar lavages (BAL) were performed using 8x1ml of ice-cold Mg- and Ca-free PBS containing 1 mM of EDTA to isolate alveolar macrophages. For this procedure, the lavages were centrifuged at 5,000 g during 10 min at 4 °C, supernatants were removed and cells pooled and washed twice in 1 ml PBS. Cells were then counted using trypan blue (the lavages contained greater than 95% of alveolar macrophages with greater than 95% viability). Finally, cells were centrifuged at 5,000 g for 10 min at 4 °C, suspended in 500 µl of cells lysis reagent (M−PER Mammalian Protein Extraction Reagent, Thermo-Scientific) and, then, kept at −20 °C. The amount of PEG40 was quantified using a PEGylated protein ELISA kit (Enzo Life Science; Farmingdale, NY, USA). This ELISA is a competitive assay specific to the backbone of PEG. It has been validated for use with a wide variety of PEG molecules including linear and branched forms as well as free and conjugated forms. The assay is more sensitive to HMW PEG molecules as PEG40. The assay, the buffer and the preparation of the samples were performed according to the manufacturer’s protocol. The PEGylated protein standard curve was prepared using the PEG40-Fab’ anti-IL17A administered to the mice. A PEGylated bovine serum albumin assay control provided in the kit was used as positive control. AM recovered from mice instilled with PBS and processed as described above were used to determine the background of the assay. The level of Fab’ was quantified by ELISA using IL17A-coated plates (Freches et al., 2017).

### 2.7 In vivo assessment of PEGylated Fab’ and PEG 40-related toxicity

To assess the toxicity profile of PEGylated Fab’ delivered by intratracheal instillation, acute and chronic administration protocols were tested. All protocols used in this study were approved by the animal ethical committee from the University of Liege and were described in accordance with the institutional guidelines for animal care (protocol references: #1001). Food and water were provided *ad libitum*.

#### 2.7.1 Acute exposure protocol

Female NMRI mice, 8-week old, were purchased from Janvier Labs (France). To assess the acute toxicity of test-compounds, mice were challenged either a) by a single intratracheal instillation or b) by three instillations on days 1, 3 and 6 of Fab’, PEG40–Fab’ or PEG40 (200 μg or 4.2 nmol/25 μl per administration, n = 8). In the placebo group, mice were treated intratracheally with PBS. Mice were sacrificed by cervical dislocation a) 24 and 72 h after the single dose instillation or b) 24 h and 7 days after the last treatment.

#### 2.7.2 Chronic exposure protocol

Mice (8-week old) were intratracheally challenged with test-compounds (*vide supra*) once per week during twelve consecutive weeks (200 μg or 4.2 nmol/25 μl per administration, n = 8). Mice were sacrificed 7 days after the last exposure by cervical dislocation.

After sacrifice, to collect BAL, a cannula was inserted into the trachea and 4 × l ml of PBS-EDTA 0.05 mM were injected into the lungs (Calbiochem, Darmstadt, Germany). Collected BAL fluid was centrifuged for 10 min at 4 °C and supernatants were stored at −80 °C for further assessments. Cell pellets were suspended in 1 ml PBS-EDTA 0.05 mM to proceed with total and differential cell counts. For differential cell counts, cells were cytocentrifuged, stained with Diff-Quick (Dade) and were classified based on morphological criteria. A total of 300 cells per slide was counted.

After BAL collection, the thorax was dissected and the left lung was insufflated with 4% paraformaldehyde and embedded in paraffin for further histological analysis. A haematoxylin-eosin staining was performed on histological lung slides allowing the assignment of a peribronchial inflammation score by observers blinded to study details (Cataldo et al., 2002). Hence, a value from 0 to 3 was adjudged to each bronchus, depending on the extension of inflammation. A score of 0 was attributed to bronchi without inflammation, a score of 1 corresponded to occasional inflammatory cells observed around bronchi, while a score of 2 represented bronchi surrounded by one to 5 layers of inflammatory cells. A value of 3 was ascribed when bronchi were surrounded by a thick layer composed of more than 5 inflammatory cells. Eight bronchi per lung sample were analysed.

ELISA assays measuring IL13 and KC protein levels in BAL supernatants were performed following protocols of Duoset ELISA development kits (R&D Systems-Biotechne). IL-6 and TGFβ1 levels in BAL supernatants were determined by custom-made ELISAs (Vink et al., 1990, Uyttenhove et al., 2011). To evaluate the presence of an increased vascular permeability, the quantitative determination of albumin in BAL fluids was performed using a Mouse Albumin ELISA kit (Alpha Diagnostic International), following manufacturer’s protocol.

To evaluate the presence of cell or tissue toxicity due to antibody-instillation, a colorimetric LDH assay was performed on 50 µl BAL supernatants following the protocol’s guidelines (Abcam).

### 2.8 Statistical analysis

Statistical analysis was performed by using GraphPad Prism software. ANOVA tests with Dunnett or Bonferroni post-tests were performed to assess statistical differences between experimental groups. *p < 0.05; **p < 0.01; ***p < 0.001.

## 3 Results

### 3.1 Evaluation of the fate of PEG40- and Fab’- moieties of PEG40-Fab’ anti-IL17A captured by alveolar macrophages

It was previously shown that alveolar macrophages captured both PEGylated and non-PEGylated Fab’ (PEG40-Fab’ anti-IL13) (Koussoroplis et al., 2014).

**Fig. 1:**
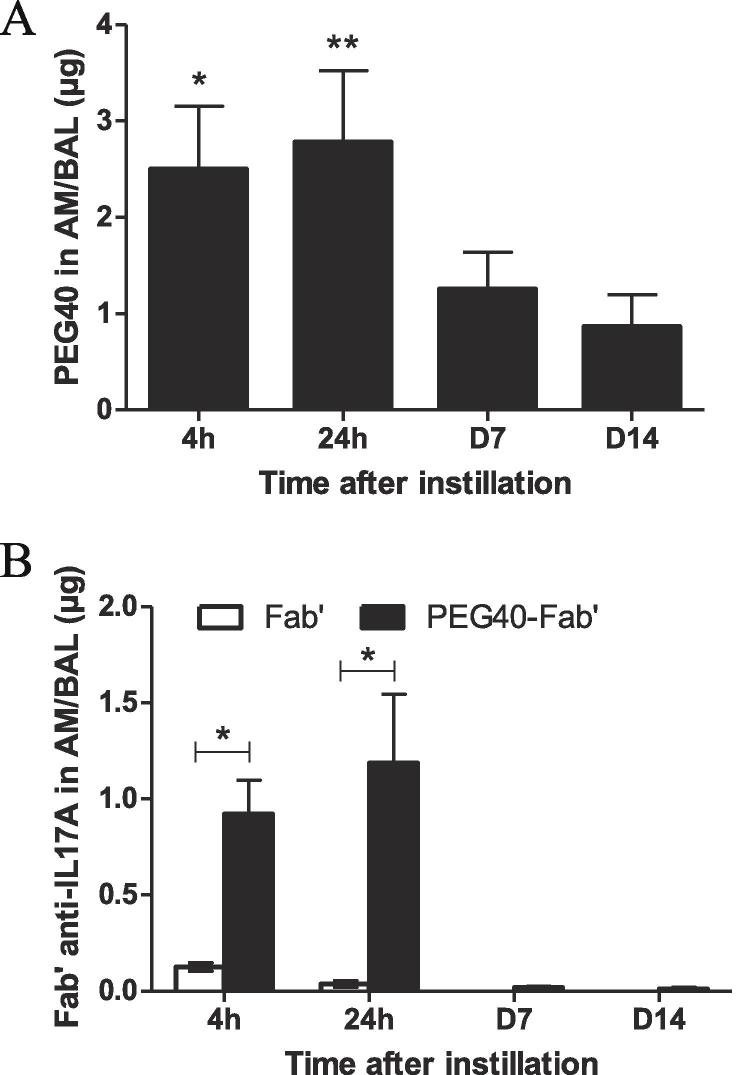
A. Quantification of PEG40 levels recovered from alveolar macrophages 4 h, 24 h, 7 days and 14 days following intratracheal delivery of PEG40-Fab’ (100 µg or 2.1 nmol) in mice. The background of the assay was determined using alveolar macrophages recovered from mice instilled with PBS. It was worth 0.085 ± 0.03 µg. Significant differences between the PEG40-treated groups and the assay background (*p < 0.05; **p < 0.01) are shown 4 and 24 h after intra-tracheal administration of PEG40-Fab’. B. Quantification of Fab’-moiety levels in alveolar macrophages 4 h, 24 h, 7 days and 14 days after intratracheal delivery of Fab’ or PEG40-Fab’ (100 µg or 2.1 nmol) in mice. The background of the assay was determined using alveolar macrophages recovered from mice instilled with PBS. It was worth 0.007 ± 0.004 µg. Significant differences between Fab’ and PEG40-Fab’ are shown (*p < 0.05). The data represent mean values ± SEM of ≥ 5 mice.

Here, the fate of the PEG40 and Fab’-moieties of the PEG40-Fab’ anti-IL17A inside alveolar macrophages was evaluated separately over 14 days. For that purpose, 100 µg of PEG40-Fab’ were intratracheally administered to NMRI mice and the amount of PEG40 contained inside alveolar macrophages was quantified. Interestingly, the PEG40-moiety of the PEG40-Fab’ was already detected in alveolar macrophages 4 h (*p < 0.05) post-delivery and its levels remained stable up to 24 h (**p < 0.01) post-administration. The amounts of PEG40 measured in alveolar macrophages decreased at 7 and 14 days post-administration suggesting that PEG40 is progressively cleared from the respiratory tract (Fig. 1A). In addition, the levels of the Fab’-moiety of PEG40-Fab’ remained stable over 24 h (Fig. 1B), as well as the levels of the PEG40-moiety (Fig. 1A). However, the levels of the Fab’-moiety were 2 times lower than the PEG40-moiety levels suggesting a faster clearance (Fig. 1A-B).

Finally, the amounts of the Fab’moiety contained inside alveolar macrophages were compared for the PEGylated and non-PEGylated Fab’ over 14 days. One hundred µg of non-PEGylated Fab’ were intratracheally administered to NMRI mice and the amount of Fab’ contained inside alveolar macrophages isolated from the BAL was quantified at 4 h, 24 h, 7 days and 14 days post-administration, as was already performed for the PEGylated Fab'. The amount of Fab’ recovered was detectable but significantly decreased at 4 h (*p < 0.05) and 24 h (*p < 0.05) in absence of a PEG40-moiety. No Fab’ conjugated or not with PEG40 were found after 7 and 14 days (Fig. 1B).

These results show that pulmonary clearance of PEGylated Fab’ orchestrated by alveolar macrophages is delayed as compared to clearance of non-PEGylated Fab’.

### 3.2 In vitro evaluation of the potential cytotoxicity induced by PEG40

In order to evaluate the potential cytotoxicity mediated by PEG40 on macrophages, cultured J774A.1 cells were exposed to high concentrations of PEG40 (1, 5 and 10 mg/ml). These PEG concentrations were selected on the basis of PEG amounts that might be administered to humans. Participants of a first-in-human study received up to 20 mg of VR942, a novel anti-human-IL13 Fab’ monoclonal antibody fragment formulated as dry-powder for inhalation (UCB Pharma, Belgium) (Burgess et al., 2017), which corresponds to 0.7 mg of Fab’ per ml of epithelial lining fluid (ELF), assuming an approximate ELF volume in humans of 30 ml (Fernandes and Vanbever, 2009). The MW of the Fab’ is 47 kDa which is similar to the MW of the PEG40 (40 kDa), and only one molecule of PEG40 is conjugated to one Fab’. It is therefore expected that approximately 20 mg of PEG40 (corresponding to 0.7 mg/ml of ELF) would be administered as well. However, this PEG40 amount might be even lower than 20 mg because PEGylation of the anti-IL17A Fab′ enhanced its intrinsic inhibitory potency nine-fold and prolonged its residence time from<24 h to more than 48 h in animal lungs *in vivo* (Freches et al, 2017).

**Fig. 2:**
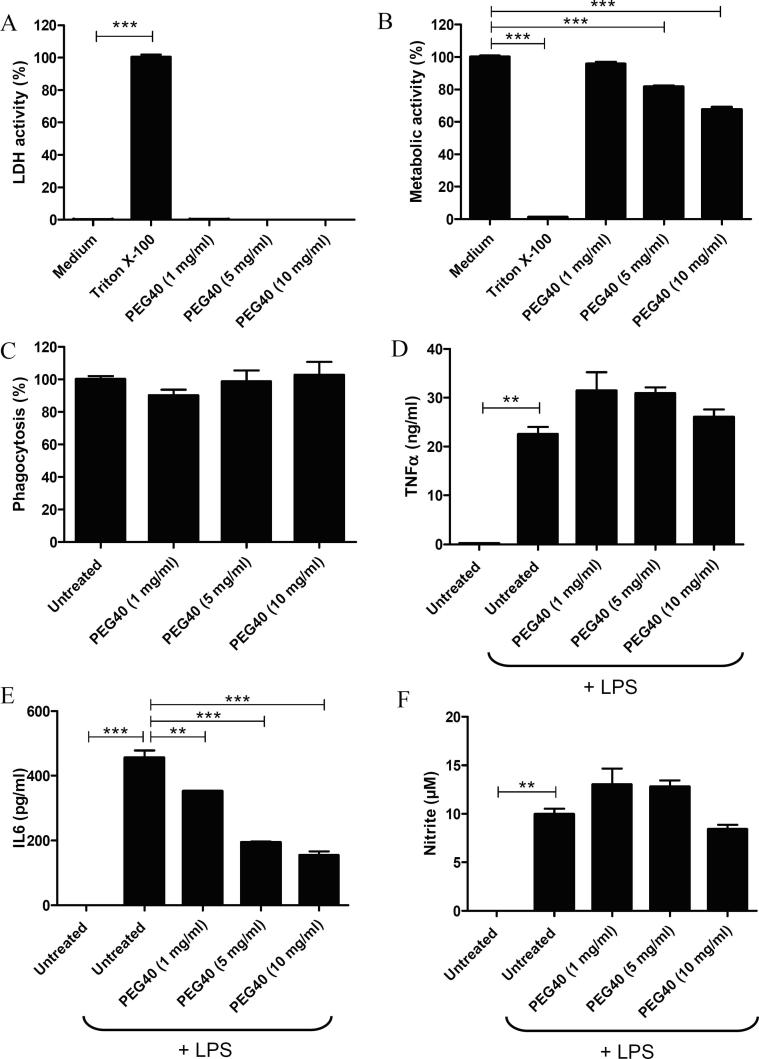
A. LDH levels measured in supernatants of J774A.1 macrophages exposed to PEG40 for 72 h. Results are presented as % from control Triton X-100-treated cells (medium) (*** p < 0.001; medium *versus* Triton X-100). B. MTT-cell metabolism assessment of J774A.1 macrophages exposed to PEG40 for 72 h Results represent mean ± SEM (n = 4) and are presented as % from control untreated cells (***p < 0.001; medium *versus* experimental groups). Data are representative of three independent experiments. C. Phagocytic activity assessment of J774A.1 cells treated with different doses of PEG40 during 3 days. Data are presented as a percentage of the phagocytic activity of untreated J774A.1 cells. Data represent the mean ± SEM (n = 4) and are representative of 3 independent experiments. D-F. Measurements by ELISA of TNFα, IL6 levels and nitrite release when J774A.1 cells were cultured in presence of LPS. Data represent the mean ± SEM (n = 2) and are representative of three different experiments (**p < 0.01; ***p < 0.001 *versus* cells incubated with LPS).

Following exposure to PEG40 for 3 days, plasma membrane integrity was evaluated by measuring LDH activity in culture medium. Except for Triton X-100 (***p < 0.001), used as a positive control for the induction of cell membrane impairment, PEG40 (up to 10 mg/ml) did not induce LDH release suggesting that the PEG40 does not induce significant damages to macrophage plasma membrane (Fig. 2A).

Cell metabolic activities were assessed with the MTT assay. Only Triton X-100-treated cells, used as positive control, showed strongly impaired metabolic activities (***p < 0.001), while untreated cells (medium) were considered to have an unimpaired (100%) metabolic activity. Interestingly, while the metabolic activity of J774A.1 cells was not impaired by the lowest concentration of PEG40 (1 mg/ml), J774A.1 metabolic activity significantly decreased when PEG40 concentration increased to 5 and 10 mg/ml (Fig. 2B, ***p < 0.001). These results indicate that macrophages treated with moderate to high doses of PEG40 display a dose-dependent moderate alteration of their metabolic activity.

### 3.3 Assessment of macrophage functional activity (phagocytosis, cytokine and nitrite production)

Potential effects of PEG40 on the functional activity of macrophages were investigated by evaluating their phagocytosis capacity. For this, J774A.1 cells were treated with different concentrations of PEG40 and incubated with fluorescent polystyrene beads. Percentages of beads phagocytosed by macrophages were equivalent in both treated and untreated macrophages suggesting that PEG40 did not impair the phagocytic activity of macrophages (Fig. 2C).

In order to validate macrophage’s functional activity, the *in vitro* responses to LPS stimulation were evaluated by measuring levels of TNFα, IL6 and nitrite in macrophage culture supernatants. TNFα and IL6 are important mediators of the immune response. Both cytokines are secreted by activated-macrophages during acute phase response. Nitrite is a marker of nitric oxide production acting as an important effector molecule in activated-macrophages. As expected, LPS treatment induced the production of TNFα (**p < 0.01), IL6 (***p < 0.001) and nitrite (**p < 0.01) by untreated J774A.1 cells. PEG40 pre-treatment did not impair the production of TNFα and nitrite, when compared to cells only challenged by LPS (untreated). However, in cells pre-treated with PEG40, IL6 production decreased significantly in a dose-dependent manner as compared to untreated cells (1 mg/ml **p < 0.01; 5 and 10 mg/ml ***p < 0.001) (Fig. 2D-F).

### 3.4 In vivo acute toxicity studies – Single dose administration

In order to evaluate the toxicity of PEG40-Fab’ conjugates and non-conjugated PEG40, 200 µg of PEG40-Fab’ anti-IL17A (10-fold the active dose as described by Koussoroplis SJ et al, 2014), PEG40 or Fab’ anti-IL17A were administered to NMRI mice by the intratracheal route. PBS was administered as a control condition. In experiments designed to assess the potential acute toxicity of PEG40, mice were exposed to a single dose and sacrificed after 24 h or 72 h to assess the recruitment of inflammatory cells to the lungs.

**Fig. 3:**
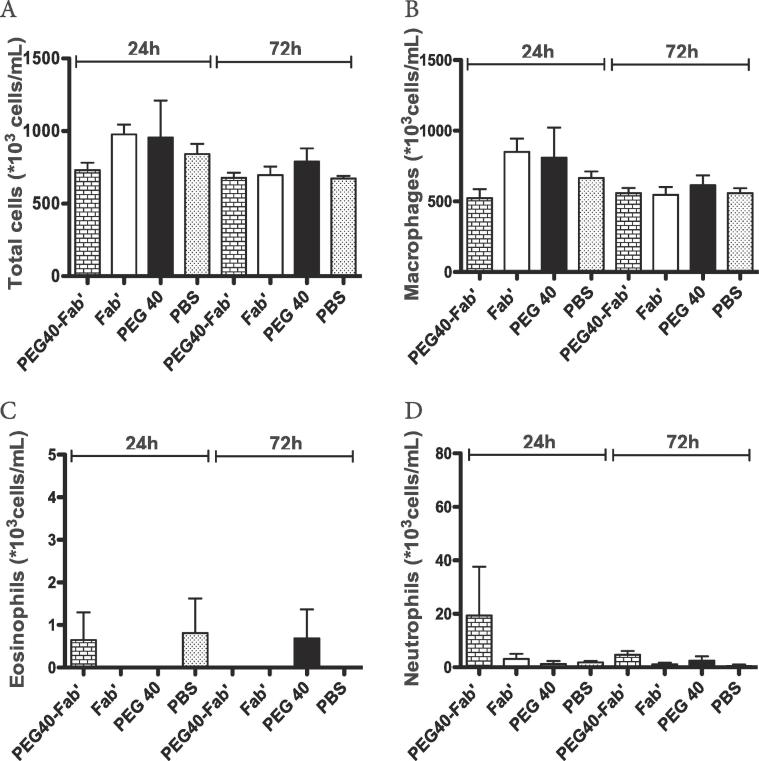
In vivo assessment of potential toxicity 24 and 72 h after a single intratracheal instillation of PEG40-Fab’, Fab’, and PEG40 (200 μg or 4.2 nmol/25 µl, n = 8 mice/experimental group). PBS was used as control treatment. Cell counts were performed in BAL after sacrifice A. Total cells, B. macrophages, C. eosinophils and D. neutrophils.

**Fig. 4:**
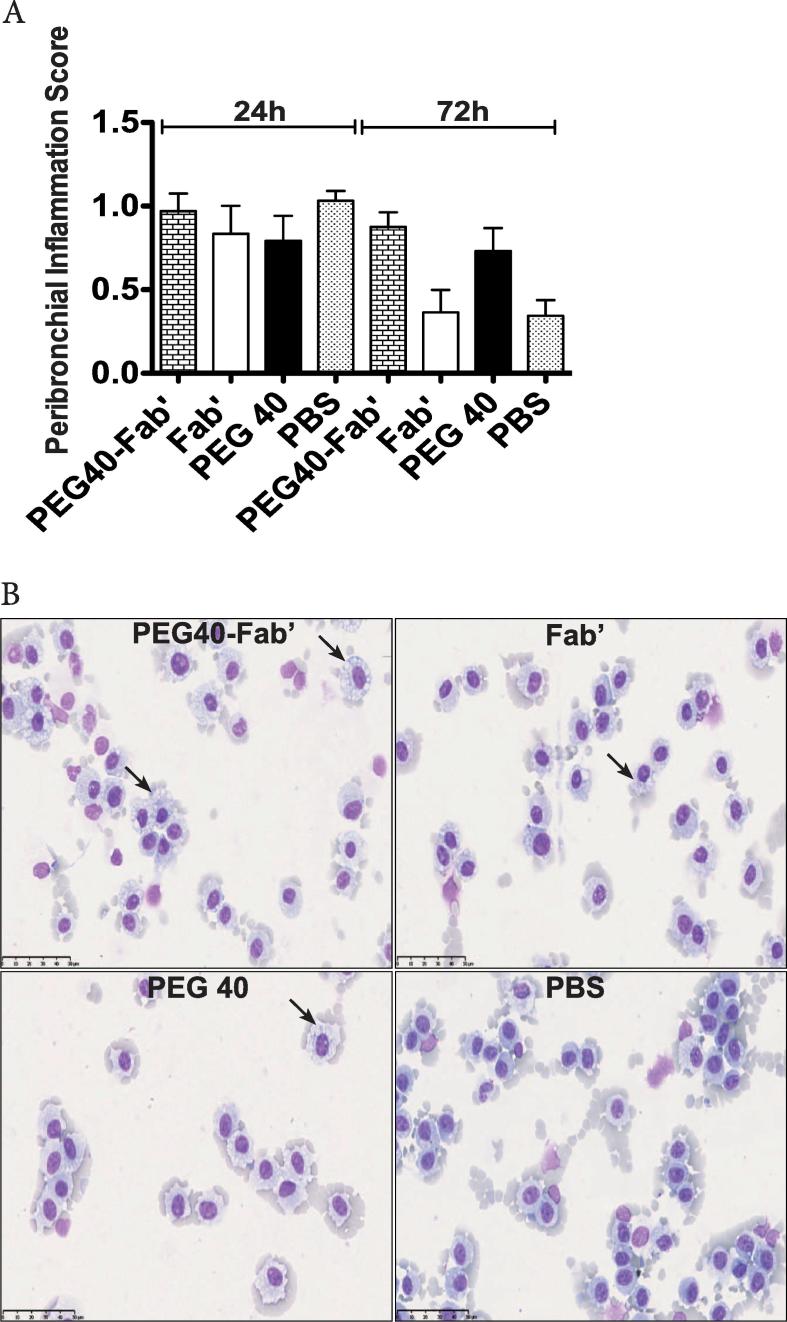
A. Assessment of the peribronchial inflammation score on histological lung slides. B. Representative photographs of BAL of mice treated with PEG40-Fab’, Fab’, and PEG40 or PBS. Foamy macrophages are marked by black arrows. Magnification 2400x. Scale bar: 50 µm. Data are presented as mean ± SEM and a representative of two experiments performed individually.

No significant differences in total cell counts, macrophages, neutrophils or eosinophils absolute values were observed in BAL between experimental groups (Fig. 3A-D). However, a non-significant increase of neutrophil numbers was observed when the PEG40-Fab’ preparation was administered, which might be explained by the measurement of low levels of endotoxin in this preparation (7.8 ng LPS/25 µl) (Fig. 3D). The peribronchial wall inflammation was evaluated and no score above 1 could be noted, suggesting that no tested component induced a pulmonary inflammatory reaction (Fig. 4A).

Animals exposed to PEG40, PEG40-Fab’ or Fab’ displayed limited amounts of foamy macrophages in the BAL fluid (Fig. 4B). These foamy alveolar macrophages were previously reported as markers of clearance of particulates from the lungs (Nikula et al, 2014).

Altogether, these data indicate that PEG40-Fab’ anti-IL17A and PEG40 alone do not induce significant pulmonary toxicity when intratracheally administered as a single-dose.

### 3.5 In vivo toxicity studies – Multiple dose administration

**Fig. 5:**
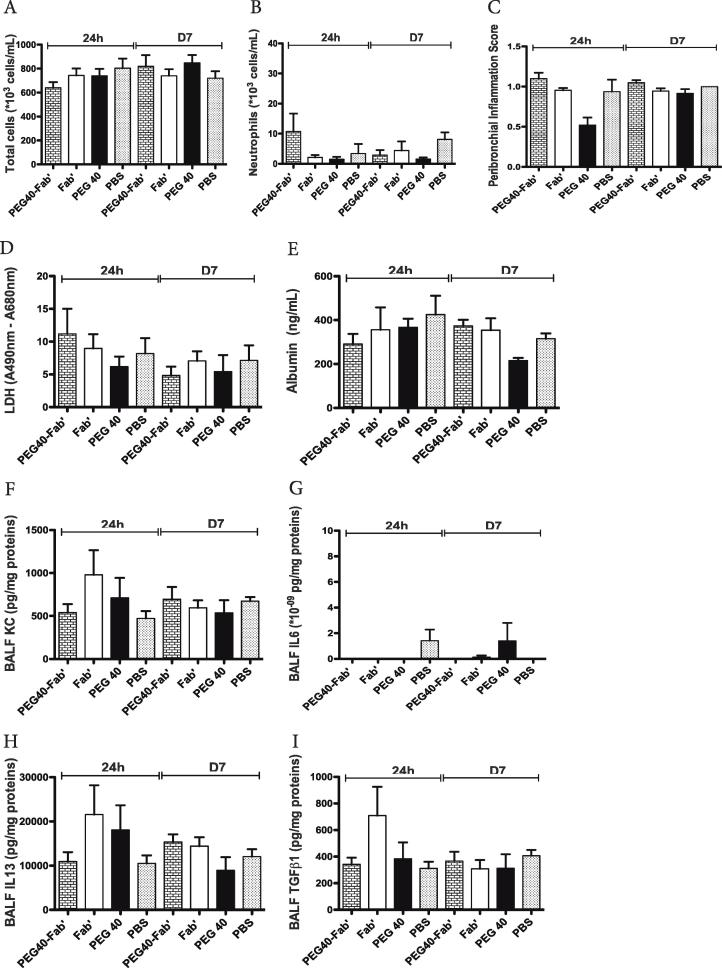
In vivo assessment of toxicity after administrations on days 1, 3 and 6 of PEG40-Fab’, Fab’ and PEG40 or PBS (200 μg or 4.2 nmol /25 µl). Measurements were performed 24 h and 7 days after the last instillation (n = 8 mice/experimental group). PBS was used as a control treatment. A-B. BAL total and differential cell counts. C. Peribronchial inflammation score measured on haematoxylin-eosin stained lung sections. Scores presented are the mean of 8 bronchi analyzed per mouse. D. LDH levels measured in BAL fluids. E. Mouse Albumin levels measured in BAL fluids. F-I. ELISA measurements of key inflammatory cytokines (KC, IL6, IL13, TGFβ1) in BAL fluids. Data are presented as mean ± SEM.

To verify that multiple administrations do not cause any harm, PEG40-Fab’ anti-IL17A, Fab’ anti-IL17A or PEG40 alone were intratracheally administered on days 1, 3 and 6. PBS was administered as a control condition. Twenty-four hours and 7 days after the last treatment, no significant difference in BAL total cell counts was observed (Fig. 5A). While macrophage or eosinophil numbers were similar between experimental groups (data not shown), a non-significant increase in neutrophil counts could be measured in PEG40-Fab’ -treated mice 24 h after the last treatment (Fig. 5B). This slight (non significant) increase can be attributed to the low endotoxin levels detected in this preparation as already stated in the short exposure study (0.78 ng LPS/25 µl). In the meantime, no detectable LPS was measured in PEG40 and Fab’ preparations. Peribronchial inflammation score revealed an average score around 1 in all experimental groups, indicating that a very slight peribronchial inflammation was present among pulmonary tissues (Fig. 5C), independent of the treatment administered and possibly related to repeated intratracheal instillation procedures.

In order to exclude any tissue toxicity or increased vascular permeability, LDH and albumin levels were assessed in BAL fluids. As shown in Fig. 5, levels of LDH or albumin were similar in all experimental conditions (24 h or 7 days post-administration) (Fig. 5D-E). Levels of key inflammatory cytokines (KC, IL6, IL13 and TGFβ1) were also measured in the BAL fluid and no significant differences were found between experimental groups (Fig. 5F-I).

Overall, multiple intratracheal administrations of PEG40-Fab’ anti-IL17A or PEG40 alone over a short period did not cause any pulmonary toxicity *in vivo*.

### 3.6 In vivo toxicity studies – Chronic administration (12 weeks)

**Fig. 6:**
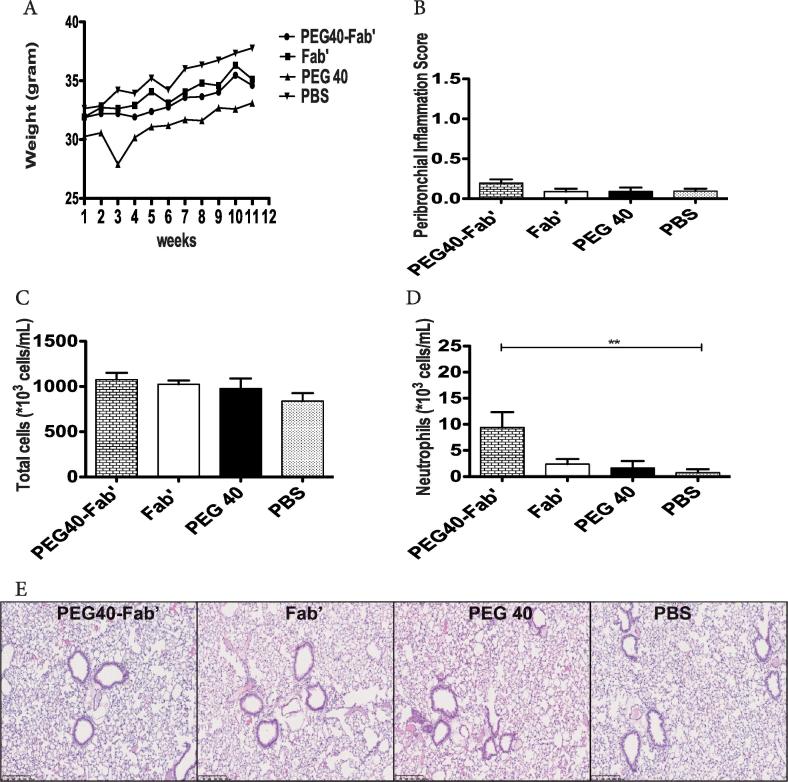
In vivo assessment of chronic toxicity after 12 weeks of intratracheal instillations on a weekly basis of PEG40-Fab’, Fab’ and PEG40 or PBS (200 μg or 4.2 nmol/25 µl per administration) (n = 8 mice/experimental group). A. Mice weight measured weekly during the chronic exposure protocol. B. Measurement of peribronchial inflammation score on lung tissues stained with haematoxylin-eosin (scores are presented as a mean of 8 bronchi per mouse). C. Total cells counts in BAL performed seven days after the last treatment. D. Neutrophil counts in BAL performed seven days after the last treatment. **p < 0.01. E. Representative microphotographs of lung tissues. Magnification 400x. Scale bar: 250 µm.

**Fig. 7:**
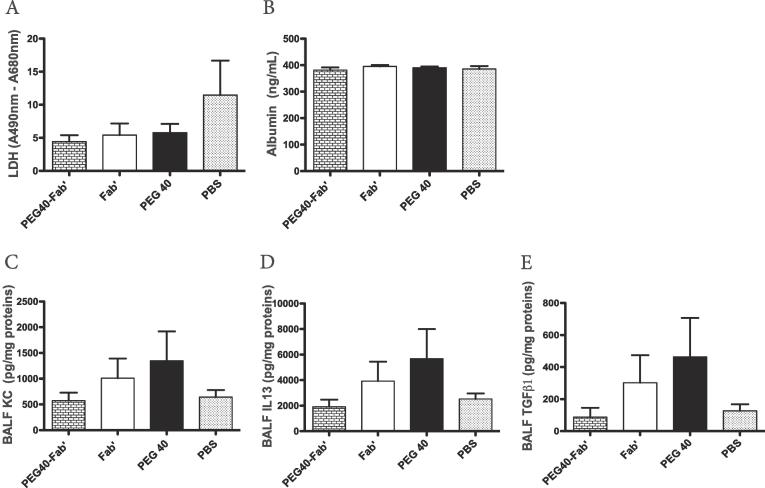
Measurements of LDH, albumin (A-B) and key inflammatory cytokines (KC, IL13, TGFβ1) by ELISA in the BAL fluid (C-E). Data are presented as mean ± SEM and are representative of two experiments performed individually.

To assess the potential chronic toxicity**,** mice were intratracheally exposed to one administration per week of PEG40-Fab’ anti-IL17A, Fab’ anti-IL17A, PEG40 alone or PBS during 12 consecutive weeks. After 12 weeks of treatment, mice did not display any significant difference in weight gain (Fig. 6A). Although mice treated with PEG40 displayed a lower weight at the time of inclusion in the experimental protocol, their growth evolution was similar to other groups (Fig. 6A). The peribronchial inflammation score excluded any sign of inflammation in bronchial walls since very low scores were measured in all experimental groups (Fig. 6B). In BAL, total cell numbers were also similar in all groups (Fig. 6C) while neutrophil numbers were higher in PEG40-Fab’ -exposed mice when compared to BAL of control PBS-treated groups (**p < 0.01; Fig. 6D). This slight neutrophilic inflammation can be the consequence of low LPS levels measured in this formulation (1.2 ng/25 µl). No significant differences in macrophage or eosinophil numbers were recorded (data not shown). These results indicate that chronic intratracheal administrations of PEG40-Fab’ is not linked to inflammatory cell recruitment to lung tissues other than a low number of neutrophils driven par LPS. Representative lung tissue photographs confirm that peribronchial inflammation after intratracheal delivery of test compounds was very limited and almost not measurable. Nevertheless, there is no evidence of test material-related lung inflammatory changes as all changes are comparable to those observed in PBS group (Fig. 6E). To get further insights into potential tissue toxicity, LDH levels were quantified in BAL samples while vascular permeability was evaluated by assessing albumin in BAL fluids (Fig. 7A-B). Moreover, key cytokines involved in a pro-inflammatory response such as KC, IL13 and TGFβ1 were measured in BAL fluids (Fig. 7C-E). The mean values for these above-cited cytokines are higher in the Fab’ or PEG only treated mice compared to PBS or PEGylated Fab’-treated groups. However, these differences are not significant and are caused by the large inter-animal variability. Levels of IL6 were undetectable in analyzed samples (data not shown). Altogether, these data suggest an absence of tissue toxicity or active inflammation in all experimental conditions (Fig. 7C-E).

## 4 Discussion

Only few studies have been conducted so far to assess the safety and potential impact on the pulmonary tissue of inhaled PEG or PEGylated compounds. Klonne *et al*. have reported a relatively low toxicity after inhalation of LMW PEGs (3.35 Da) in a two-week aerosol inhalation study in rats (Klonne et al., 1989). More recently marketed liquids dedicated to inhalation with e-cigarettes and containing various LMW PEGs (mainly 400 Da PEG) have been widely used. Although a systematic placebo-controlled study of potential side effects of the inhalation of PEGs found in e-cigarettes was not conducted, the overall toxicity relying on the use of these devices appears acceptable (Chun et al., 2017). While LMW PEGs (<10 kDa) are considered safe, the potential toxicity of inhaled HMW PEGs (as up 40 kDa for biopharmaceuticals) remains to be evaluated. The aim of the present study was therefore to evaluate the potential toxicity after pulmonary administration of a PEGylated Fab’ anti-IL17A and, by extension, of a 2 armed-PEG40, a HMW PEG (40 kDa).

Alveolar macrophages are the first and most exposed cells to inhaled compounds and they play a key role in immune surveillance by removing bacteria, foreign bodies or other materials from the lungs. Previous results have already demonstrated the uptake of PEGylated Fab’ anti-IL13 (Koussoroplis et al., 2014) or PEG up to 20 kDa (Gursahani et al., 2009) by these cells after pulmonary administration. Moreover, after intravenous administration in rats, unconjugated-PEG40 detected in the lungs were localized inside alveolar macrophages (Ivens et al., 2015). Therefore, we studied PEG40 retention inside alveolar macrophages and the potential impact on cell functions. Similarly to Koussoroplis et al. (Koussoroplis et al., 2014)*,* we found that alveolar macrophages captured PEG40-Fab’ anti-IL17A after a single pulmonary administration. The amount of the Fab’-moiety measured inside these cells was 2-fold lower as compared to the amount of the PEG40 moiety, suggesting its faster elimination. This might be explained by a higher propensity to proteolysis of the protein part (Fab’) while PEG40 appears as less easily biodegradable. In addition, when unPEGylated Fab’ was delivered, 10-fold lower amount of Fab’ was found in macrophages. This might be explained by an enhanced proteolytic resistance of the Fab’ moiety in PEG40-Fab’ provided by PEG (Chapman, 2002, Lee et al., 2009, Youn et al., 2007, Youn et al., 2008, Zhang et al., 2014). An alternative explanation could be that alveolar macrophages were exposed to higher local concentrations of PEG40-Fab’ as compared to non-PEGylated Fab’ because of the prolonged residence time in the airspace of the PEGylated Fab’ (Freches et al., 2017, Koussoroplis et al., 2014).

The amounts of PEG40 found inside murine alveolar macrophages remained stable over 24 h but only low levels of PEG40 were detected in murine alveolar macrophages at 7 days post-administration suggesting that the PEG-moiety is progressively eliminated from the alveolar macrophages at some point between 1 and 7 days after a single exposure. By contrast, Gursuhani *et al.* have shown that following intratracheal delivery in rats, LMW PEG (<2 kDa) were cleared from the lungs within 48 h while HMW PEG (20 kDa) remained in the lung cells and tissues for up to 7 days (Gursuhani et al., 2009). Details of the fate of the PEG once ingested by phagocytic cells are not fully understood yet. Metabolism of PEG in phagosomes and lysosomes is likely to be limited since the enzyme repertoire for PEG metabolism in the body is restricted (Baumann et al., 2014, Caliceti and Veronese, 2003, Ivens et al., 2015, Webster et al., 2007). Accordingly, Moyers *et al.* have recently shown that basal insulin peglispro (BIL), a novel PEGylated insulin lispro is internalised by human 293HEK cells and localised within early endosomes. While the insulin is degraded inside the cell, the PEG-moiety is recycled out of the cell without being catabolized (Moyers et al., 2017). Absorption of PEG (up to 20 kDa) from the lungs into the systemic circulation has also been demonstrated (Gursuhani et al., 2009). In addition, urinary excretion has been reported as a major elimination route for PEG moieties of <50 kDa (Webster et al., 2007, Yamoaka et al., 1994). As suggested by Patil et al., following pulmonary administration of PEG40-Fab’ anti-IL17A, the mucociliary clearance could be an alternative pathway of elimination (Patil et al., 2018). Nevertheless, while the mechanisms of uptake and recycling out of the cells are not fully understood, we bring *in vitro* and *in vivo* evidence indicating that the integrity of the macrophage membranes in not altered as assessed by the low LDH levels released following incubation of macrophages with PEG40 and low LDH levels measured in the BAL of mice treated with PEG40-Fab’ anti-IL17A. However, further investigations are necessary to evaluate the potential accumulation and related effects of PEG40 inside alveolar macrophages after repeated administration of PEG40-Fab’.

The main functions of macrophages are the removal of foreign particles and the control of pathogens, as well as the regulation of local pulmonary immune responses by their secretions of cytokines and microbicide agents (as IL6, TNFα or NO). In our study, PEG-associated effects on macrophage function were evaluated and showed no significant alterations of the phagocyte functions of macrophages after *in vitro* incubation with PEG40. This is in line with the studies conducted to support the development of Cimzia, a PEGylated-Fab’ anti-TNFα. These studies reported only a modest suppression of *in vitro* macrophages-associated phagocytic activities despite vacuolization of several cell types (EMEA/664021/2009; 2009). However, a significant dose-dependent decrease in IL6 production was observed when LPS-stimulated macrophages were incubated with PEG40 suggesting an anti-inflammatory activity for PEG40 on alveolar macrophages. This unexpected anti-inflammatory property was also described by Ackland *et al.* who describe that LMW PEG reduced inflammatory cytokine expression, pyrexia and mortality by greater than 50% in both LPS and zymosan models of sepsis. Moreover, LMW PEG was reported to reduce cytokines expression both *in vivo* and *in vitro*, and to attenuate human neutrophils activation in response to LPS or zymosan. However, according to their observations, HMW PEG did not exhibit such profound anti-inflammatory effects (Ackland et al., 2010). The impact of the decreased IL-6 levels on protection against infection will need to be determined.

Finally, our *in vivo* experiments established that one single or repeated (three doses two to three days apart or 1 dose/week for 12 weeks) administration via the intratracheal route of PEG40-Fab’ anti-IL17A did not induce any significant pulmonary toxicity in mice. Two hundred µg of PEG40-Fab’ were administered to mice corresponding to 10-fold the active dose as established by Koussoroplis *et al.* (Koussoroplis et al, 2014). Once to twice weekly administration of the PEG40-Fab’ anti-IL17A might represent the dosing regimen required for therapy in humans. In fact, drug clearance is known to be slower in humans than in rodents (Lee at al., Drug Des Devel Ther, 12: 495–504, 2018). In line with this, the PEG40-Fab’ anti-IL17A has been shown to remain present in the lungs for more than 2 days in mice, rats and rabbits with a tendency of longer residence time in higher species (Freches et al, 2017). However, when mice received the PEG40-Fab’ conjugates, neutrophil counts were slightly but non-significantly elevated in the BAL. This very mild increase in neutrophil counts is likely to be caused by detectable levels of LPS that contaminated the PEG40-Fab’ preparation during the manufacturing process reaching levels slightly higher than those recommended for an inhalation formulation (Malyala, 2008). Nevertheless, no significant pulmonary histologic inflammation or changes in secretion of inflammatory markers were observed after acute or chronic intratracheal administrations of the HMW PEG40 or PEG40-Fab’ whereas vacuolated alveolar macrophages were observed after only one single dose in the BAL. Cellular vacuolization is a common finding after administration of PEGylated biopharmaceuticals or unconjugated-PEG, in animals and in humans (Bendele et al., 1998, Ivens et al., 2015, Rudmann et al., 2013, Young et al., 2007, Webster et al., 2007). However, many authors claim that vacuolization of alveolar macrophages, including vacuolization induced by PEG internalisation, is an adaptive response to an increased demand for PEG clearance rather than a toxic effect of PEG on cellular physiology. Moreover, PEG-associated vacuolization in macrophages is partially or completely reversed after a treatment-free period, given sufficient recovery time (Hoffman et al., 2015, Ivens et al., 2015, Rudmann et al., 2013). Interestingly, it should be noted that unPEGylated Fab’ also induced vacuolization of alveolar macrophages highlighting the fact that vacuolization is not specific to the PEG and can occur in response to different stimuli. Indeed, vacuolated macrophages are considered as a normal physiological sequelae caused by normal pulmonary clearance mechanisms following inhalation of pharmaceutical materials (Nikula et al, 2014). However, uncertainties remain about the impacts of vacuolization, particularly for long-term administrations. Nevertheless, registered PEGylated proteins administered systemically (e.g. Cimzia) induce vacuolization without serious adverse event reported.

Taken together, our results provide indications for a favourable safety profile for PEG40 or PEG40-Fab’ anti-IL17A administered by the intra-tracheal route and therefore suggest that no obvious toxicity is expected when these compounds are given by inhalation. Moreover, we show that pulmonary-administered PEG40 do not harm macrophages and are progressively cleared from alveolar macrophages suggesting that an accumulation in the lungs should be monitored in case of chronic administration, as intended for PEGylated antibody treatment, regarding the time allowed between consecutive doses. Additional studies in rodents and non-rodents using GLP-produced PEGylated antibodies are needed before reaching the conclusion that HMW PEG inhalation is completely safe. This step will be mandatory before starting any clinical trial. However it is more likely that the activity of the protein conjugated to the PEG will define the tolerability and risk profile in a clinical population.

## 5 Funding information

This work was supported by the WALEO3 program of the Walloon Region, Belgium [Grant 816862]. Complementary funding was provided by grants from The Fondation Leon Fredericq (Liège University and CHU Liege).

The funding source(s) had no involvement in study design; in the collection, analysis and interpretation of data; in the writing of the report; and in the decision to submit the article for publication.
